# Clinical and Ophthalmological Characteristics and Therapeutic Management of Patients With Sarcoidosis

**DOI:** 10.7759/cureus.93898

**Published:** 2025-10-05

**Authors:** Karla I Llerenas-Aguirre, Bruno Taboada Moreno, Luis P Orozco Gómez

**Affiliations:** 1 Ophthalmology, National Autonomous University of Mexico, Mexico City, MEX; 2 Ophthalmology, Centro Médico Nacional 20 de Noviembre, Mexico City, MEX; 3 Molecular Medicine, University of Colima, Colima, MEX; 4 Uveitis and Ocular Inflammation, Centro Médico Nacional 20 de Noviembre, Mexico City, MEX; 5 Inflammatory Eye Disease Clinic, Asociacion Para Evitar la Ceguera en México Hospital Dr. Luis Sánchez Bulnes, Mexico City, MEX; 6 Retina and Vitreous Department, Centro Médico Nacional 20 de Noviembre, Mexico City, MEX

**Keywords:** epidemiology, granuloma, granulomatous disease, mexico, ocular inflammation, ocular sarcoidosis, sarcoidosis, uveitis

## Abstract

Objective

This study aimed to describe the clinical and ophthalmological characteristics, therapeutic management, and visual outcomes of patients with sarcoidosis at a tertiary referral center in Mexico over 10 years.

Patients and methods

This retrospective case series included patients diagnosed with sarcoidosis between 2015 and 2025 at Centro Médico Nacional “20 de Noviembre.” Diagnosis was based on clinical, radiological, and histopathological criteria aligned with the World Association of Sarcoidosis and Other Granulomatous Disorders (WASOG) and the International Workshop on Ocular Sarcoidosis (IWOS) guidelines. Patients with complete records and at least one ophthalmologic evaluation were included. Data on demographics, systemic and ocular involvement, comorbidities, and treatments were collected and analyzed descriptively.

Results

Eight patients (75% female; median age: 67 years) were included, reflecting the rarity of sarcoidosis in our setting. Ocular involvement was present in five patients (62.5%). Keratoconjunctivitis sicca (KCS) was the most common manifestation, affecting four patients (50%), all bilaterally. Two patients (25%) presented with bilateral non-granulomatous anterior uveitis, one of whom progressed to panuveitis with peripheral chorioretinal lesions and uveitic macular edema. An asymptomatic solitary choroidal granuloma was identified in one patient (12.5%) through clinical examination and optical coherence tomography (OCT)-enhanced depth imaging (EDI). Visual acuity (VA) was assessed only in the five patients who underwent ophthalmologic evaluation; the remaining three were not assessed, as ophthalmologic consultation was not requested, likely due to the absence of ocular symptoms at presentation. Among evaluated patients, 37.5% had normal visual acuity, 25% had mild impairment, and one patient (12.5%) had severe visual loss. All patients received corticosteroids: 87.5% systemically and 25% via topical, periocular, or intravitreal routes. Immunosuppressive therapy was used in 75% (50% methotrexate, 25% azathioprine), and one patient (12.5%) received biologic therapy (adalimumab). Follow-up ranged from three to 10 years (median: five years), with two patients currently under active treatment for ocular sarcoidosis after three years of follow-up.

Conclusions

Ocular involvement was common in our cohort and, in some cases, represented the initial manifestation of sarcoidosis. KCS was the most frequently observed ocular finding. While quantitative VA data were limited, most evaluated patients maintained good visual function. Systemic corticosteroids were the mainstay of treatment, with immunosuppressants and biologics reserved for refractory cases. Given the potential for asymptomatic ocular disease, routine ophthalmologic evaluation is recommended for all patients with sarcoidosis, regardless of the presence of ocular symptoms.

## Introduction

Sarcoidosis is a chronic, multisystemic granulomatous disease of unknown etiology, histologically characterized by non-caseating granulomas [1,2]. Its clinical presentation varies widely depending on geographic and ethnic factors. Although well-documented in European, North American, and Asian populations, data from Latin America, particularly Mexico, are scarce, limiting our understanding of regional patterns and hindering the development of context-specific diagnostic and therapeutic guidelines [1,3]. The disease’s pathogenesis remains unclear, with both environmental and genetic factors playing key roles. Environmental exposures such as insecticides, beryllium, and microbial antigens, especially Mycobacterium species and Propionibacterium acnes, have been implicated [2,3]. Genetic susceptibility has been associated with certain human leukocyte antigen (HLA) class I and II alleles (HLA-B8, HLA-DRB1*1101), as well as genes like butyrophilin-like 2 and annexin A11 [2,4-7].

Sarcoidosis can affect virtually any organ system, with diagnosis typically based on clinical, radiologic, and histopathologic findings [8]. The World Association of Sarcoidosis and Other Granulomatous Disorders (WASOG) classification criteria are widely used to estimate the probability of organ-specific involvement [8,9]. Ocular involvement is a recognized feature of sarcoidosis, occurring in 10-89% of cases across studies, and may be the initial manifestation in 7-30% of patients [1,2,10-15]. Uveitis is the most frequently reported ocular finding (20-61%), although any ocular structure can be affected [1]. Early recognition of ocular signs is essential, as they may precede systemic disease and offer an early diagnostic window [2,3,10,16,17]. Treatment typically begins with corticosteroids, while immunosuppressive and biologic agents are reserved for refractory or severe cases [1,15].

Despite the high prevalence and potential severity of ocular sarcoidosis, the absence of robust epidemiological data from Latin America complicates early diagnosis and optimal care in this region [18,19]. This study endeavored to address this knowledge gap by characterizing the clinical and ophthalmological features, comorbidities, and therapeutic management of sarcoidosis in a Mexican cohort over 10 years at a tertiary referral center. Our findings aim to inform local clinical practice and contribute to the development of regionally relevant strategies.

## Materials and methods

Study design and setting

This retrospective case series was conducted at Centro Médico Nacional “20 de Noviembre”, a tertiary referral hospital in Mexico City. We reviewed the medical records of all consecutive adult patients (aged 18 years or older) diagnosed with systemic sarcoidosis between January 2015 and January 2025. Patients were identified through the hospital’s electronic database using the International Classification of Diseases, Tenth Revision (ICD-10) codes corresponding to sarcoidosis (D86), and diagnoses were confirmed by detailed chart review.

Eligibility criteria

Inclusion Criteria

Patients with a diagnosis of sarcoidosis established by the treating physician based on compatible clinical and radiological findings, supported by histopathological confirmation of non-caseating granulomas, in accordance with the diagnostic criteria of WASOG [8]; availability of a complete medical record including demographic, clinical, histopathological, and therapeutic data; and at least one ophthalmologic evaluation documented during the follow-up period. However, ophthalmologic assessment was requested only when ocular symptoms or signs were present, and thus only five of the eight patients (62.5%) underwent formal ophthalmologic evaluation. In these cases, the revised International Workshop on Ocular Sarcoidosis (IWOS) criteria were retrospectively applied to assess the likelihood of ocular sarcoidosis [20], based on intraocular findings, systemic evidence, and histological confirmation.

Exclusion Criteria

Patients were excluded if they had incomplete records, lacked histopathological confirmation, did not undergo ophthalmologic evaluation, had an uncertain diagnosis, or were followed up for less than six months. The follow-up period was defined as the time from the date of histopathological diagnosis of sarcoidosis to the date of the last documented clinical evaluation in the medical record, including outpatient visits and any other relevant clinical assessments. Of the 17 patients initially identified, nine were excluded for these reasons, resulting in a final sample of eight patients.

Assessments

Visual acuity (VA) was measured using a Snellen chart and converted to logarithm of the minimum angle of resolution (LogMAR) values. VA was classified as normal (LogMAR 0.0-0.18), mild visual impairment (LogMAR 0.30-0.54, equivalent to best-corrected visual acuity (BCVA) 20/40-20/70), moderate (LogMAR 0.54-2.40, BCVA 20/70-20/200), or severe (LogMAR >2.40, BCVA <20/200). VA data were only available for the five patients who underwent ophthalmologic examination. The remaining three patients did not have VA recorded, most likely due to the absence of ocular symptoms, although this was not explicitly stated in the clinical records.

Data collection

Demographic and clinical variables, including age, sex, systemic and ocular manifestations, comorbidities, and treatment regimens, were extracted using a standardized data collection form. Ocular findings evaluated included uveitis, uveitic macular edema, keratoconjunctivitis sicca (KCS), dacryoadenitis, and other ocular abnormalities potentially associated with sarcoidosis. KCS was diagnosed based on compatible symptoms (e.g., dryness, burning, or foreign body sensation) and at least one objective test: tear film breakup time (TBUT) <10 seconds, or positive ocular surface staining using the Oxford Schema [21].

Statistical analysis

All data were analyzed using descriptive statistics. Categorical variables were reported as frequencies and percentages, while continuous variables were summarized using means, medians, standard deviations (SD), and ranges, as appropriate. Statistical analysis was performed using IBM SPSS Statistics, version 30 (IBM Corp., Armonk, NY).

Ethical considerations

This study was conducted in accordance with the ethical principles of the Declaration of Helsinki and received approval from the Institutional Review Board (IRB) of Centro Médico Nacional “20 de Noviembre” under protocol number 455.2024. The analysis presented here corresponds to the sarcoidosis subgroup from the broader IRB-approved protocol titled “Prevalence of Ophthalmological Manifestations in Rheumatological Diseases.” All patient data were anonymized to ensure confidentiality.

## Results

Eight patients diagnosed with sarcoidosis over 10 years were identified. The median age was 67 years (IQR: 56-74); the mean age was 61 ± 17.7 years. Women represented 75% of the study cohort. Pulmonary involvement was the most common manifestation, observed in five patients (62.5%), followed by cutaneous involvement in two patients (25%), ocular involvement in two patients (25%), gastrointestinal involvement in one patient (12.5%), and cardiac involvement in another (12.5%). At disease onset, the most frequent symptom was dyspnea (50%), followed by ocular pain (25%), granulomatous dermatitis (25%), and cough (25%). Less common initial presentations included abdominal pain (12.5%) and photophobia (12.5%) (Table 1).

**Table 1:** Demographic and initial manifestations of patients with sarcoidosis (n=8) IQR: interquartile range

Characteristic	Value
Median age, years (IQR)	67 (56–74)
Sex, n (%)	
Female	6 (75%)
Male	2 (25%)
Initial manifestations, n (%)	
Dyspnea	4 (50%)
Ocular pain	2 (25%)
Granulomatous dermatitis	2 (25%)
Cough	2 (25%)
Abdominal pain	1 (12.5%)
Photophobia	1 (12.5%)

Pulmonary disease was the most frequent systemic manifestation, present in five patients (62.5%), and was primarily confirmed by intrathoracic lymph node biopsy. Cutaneous and ocular involvement were each observed in two patients (25%), while cardiac and gastrointestinal sarcoidosis were identified in one patient each (12.5%). Ocular cases were evaluated using the revised IWOS criteria, with two patients meeting the diagnostic criteria [20]. Full diagnostic methods are detailed in Table 2.

**Table 2:** Systemic involvement and diagnostic methods of sarcoidosis (n=8) IWOS: International Workshop on Ocular Sarcoidosis; SPECT: single photon emission computed tomography; PET: positron emission tomography

System involved	N (%)	Diagnostic method
Pulmonary	5 (62.5%)	Mediastinal lymph node biopsy (4); lung biopsy (1)
Cutaneous	2 (25%)	Skin biopsy
Ocular	2 (25%)	IWOS revised criteria
Cardiac	1 (12.5%)	SPECT/PET imaging
Gastrointestinal	1 (12.5%)	Mesenteric lymph node biopsy

According to the WASOG organ assessment instrument [8], pulmonary involvement was classified as “highly probable” in five patients (62.5%), cutaneous involvement as “highly probable” in two patients (25%), and ocular involvement as “highly probable” in two patients (25%). Both cardiac and gastrointestinal involvement were classified as “highly probable” in one patient (12.5%) based on compatible findings on SPECT imaging and gastrointestinal symptoms, respectively, in the context of histological confirmation of systemic sarcoidosis via mesenteric lymph node biopsy.

Ophthalmologic evaluation was requested in five of the eight patients (62.5%), who were also the only individuals assessed for distance VA using a Snellen chart. Ocular manifestations were the initial presentation in two patients (25%). Two patients presented with bilateral non-granulomatous anterior uveitis. Slit-lamp examination revealed fine anterior chamber cells (1+) and fine keratic precipitates in both cases. One patient initially presented with bilateral non-granulomatous anterior uveitis and later developed additional ocular signs, including bilateral panuveitis, multiple chorioretinal peripheral lesions, uveitic macular edema, and periphlebitis.

Although no biopsy of the parahilar lymph nodes was performed, the presence of biopsy-proven cutaneous granulomas supported the diagnosis of systemic sarcoidosis. Based on these ocular findings combined with systemic evidence, the patient fulfilled the revised IWOS criteria for ocular sarcoidosis, leading to the diagnosis of both ocular and cutaneous sarcoidosis [20]. The other patient exhibited anterior chamber cells and fine retrokeratic pigment on slit-lamp examination, but lacked characteristic features such as keratic precipitates or iris nodules, and did not fulfill any of the intraocular signs defined in the revised IWOS criteria [20]. Consequently, the patient did not meet the diagnostic criteria for ocular sarcoidosis [20]. A diagnosis of pulmonary sarcoidosis was established through biopsy of mediastinal lymph node granulomas.

In both patients diagnosed with ocular sarcoidosis, the WASOG instrument was applicable, as each had histologic confirmation of systemic sarcoidosis (cutaneous granulomas in one case and mediastinal lymph node biopsy in the other). Other potential etiologies, particularly infectious causes such as tuberculosis and syphilis, were reasonably excluded through negative serologic and microbiologic testing. In the patient whose initial manifestation was dacryoadenitis, an asymptomatic solitary choroidal granuloma was subsequently diagnosed through fundoscopic examination and optical coherence tomography (OCT)-enhanced depth imaging (EDI). Systemic involvement was further supported by a positive SPECT-CT scan and elevated serum angiotensin-converting enzyme (ACE) levels. These findings classified ocular involvement as a highly probable manifestation of sarcoidosis according to WASOG consensus criteria [8], and also fulfilled the revised IWOS criteria for ocular sarcoidosis [20].

Ophthalmic involvement was identified in five patients (62.5%), all of whom underwent formal ophthalmologic evaluation. The most frequent ocular manifestation was bilateral KCS, observed in four patients (50%). Diagnosis of KCS was based on compatible symptoms (ocular dryness, burning, or foreign body sensation) and at least one objective finding: tear film breakup time (TBUT) <10 seconds and/or positive ocular surface staining using the Oxford grading scale [21]. One of these patients also had a comorbid diagnosis of rheumatoid arthritis, which may have contributed to both the ocular surface findings and the development of bilateral non-granulomatous anterior uveitis.

Additional ocular findings included bilateral pterygium (25%), bilateral primary open-angle glaucoma (12.5%), bilateral cataract (12.5%), and unilateral corneal leukoma (12.5%) (Table 3). No cases of scleritis, peripheral ulcerative keratitis (PUK), or optic neuritis were documented during the study period.

**Table 3:** Ocular manifestations (n=8) ^*^One patient presented with anterior uveitis and progressed to panuveitis KCS: keratoconjunctivitis sicca

Ocular findings	N (%)
Ocular involvement: 5 (62.5%)	
KCS (bilateral)	4 (50%)
Uveitis: 2 (25%)	
Anterior uveitis (non-granulomatous, bilateral)	2 (25%)^*^
Intermediate uveitis (bilateral)	1 (12.5%)^*^
Panuveitis (bilateral)	1 (12.5%)^*^
Pterygium (bilateral)	2 (25%)
Uveitic macular edema (bilateral)	1 (12.5%)
Vasculitis (periphlebitis, bilateral)	1 (12.5%)
Chorioretinal peripheral lesions (bilateral)	1 (12.5%)
Choroidal granuloma (unilateral, solitary)	1 (12.5%)
Cataract (bilateral)	1 (12.5%)
Primary open-angle glaucoma (bilateral)	1 (12.5%)
Initial presentation of disease: 2 (25%)	
Dacryoadenitis (unilateral)	1 (12.5%)
Anterior uveitis (non-granulomatous, bilateral)	1 (12.5%)^*^

VA data were available for all five patients who underwent ophthalmologic evaluation. Three patients (37.5%) had normal VA in both eyes, ranging from 20/20 (LogMAR 0.0) to 20/25 (LogMAR 0.10). Two patients (25%) presented with mild visual impairment (BCVA 20/40-20/70, LogMAR 0.30-0.54); one of them (12.5%) also had severe visual impairment (BCVA <20/200, LogMAR >2.40) in one eye. Among the two patients diagnosed with uveitis, one had mild impairment at diagnosis with a VA of LogMAR 0.54 and 0.30 in the right and left eye, respectively.

Serum ACE levels were available in two patients (25%), both of whom had elevated values (median 70.6 U/L). Serum calcium levels were also available in two patients (25%), with both results within normal range (median 9.35 mg/dL). Due to the limited number of tests performed, these findings should be interpreted as anecdotal. Infectious etiologies were excluded in all patients. Tuberculosis was ruled out in all cases using the purified protein derivative (PPD) skin test. However, one patient (12.5%) developed disseminated tuberculosis one year after initiating biologic therapy with adalimumab.

Comorbidities were prevalent, with hypertension being the most frequent (50%), either isolated or in association with other conditions. Type 2 diabetes mellitus was present in two patients (25%), both of whom also had hypertension. Other comorbidities included rheumatoid arthritis with hypothyroidism (12.5%), prostate and central nervous system (CNS) tuberculosis (12.5%), breast cancer (12.5%), thyroid nodule (25%), chronic kidney disease (12.5%), and Jackson-Sertoli syndrome/pachyonychia congenita type II (12.5%). Most comorbidities were diagnosed before the diagnosis of sarcoidosis. Exceptions included Jackson-Sertoli syndrome, thyroid nodule, and prostate/CNS tuberculosis, which were identified during follow-up. One patient had hypertension in association with both cutaneous and cardiac sarcoidosis (12.5%) (Table 4).

**Table 4:** Comorbidities in the study population (n=8)

Comorbidity	N (%)
Hypertension	4 (50%)
Type 2 diabetes mellitus	2 (25%)
Thyroid nodule	2 (25%)
Rheumatoid arthritis + hypothyroidism	1 (12.5%)
Tuberculosis (disseminated)	1 (12.5%)
Breast cancer	1 (12.5%)
Chronic kidney disease	1 (12.5%)
Jackson-Sertoli syndrome/pachyonychia congenita	1 (12.5%)

All patients received corticosteroid therapy. Seven patients (87.5%) were treated with systemic corticosteroids (oral prednisone and intravenous methylprednisolone) due to systemic involvement. Two patients (25%) received topical, periocular, or intravitreal ocular corticosteroids. Notably, one patient received all three forms of corticosteroid therapy (systemic, topical ocular, and periocular injection) due to extensive multisystem and ocular involvement (Table 5).

**Table 5:** Therapeutic management (n=8) ^*^One patient switched from methotrexate to azathioprine

Treatment modality	N (%)
Corticosteroids (100%)	
Systemic (oral prednisone and intravenous methylprednisolone)	7 (87.5%)
Topical (prednisolone acetate ophthalmic)	2 (25%)
Periocular (methylprednisolone acetate)	2 (25%)
Intravitreal (dexamethasone implant)	1 (12.5%)
All routes combined (systemic + local)	1 (12.5%)
Immunosuppressive agents (75%)	
Methotrexate	4 (50%)
Azathioprine	2 (25%)^*^
Biologic therapy (12.5%)	
Adalimumab	1 (12.5%)

Immunosuppressive therapy was administered to six patients (75%). Methotrexate was prescribed to four patients (50%), while azathioprine was used in two patients (25%). One patient, initially treated with methotrexate, corticosteroids, and adalimumab (a biologic therapy), required a switch to azathioprine due to methotrexate-related side effects (Tables 5-6). 

**Table 6:** Characteristics and treatment of patients with sarcoidosis ^*^POAG: primary open-angle glaucoma as a medical history finding OD: right eye; OS: left eye; F: female; M: male; KCS: keratoconjunctivitis sicca; MTX: methotrexate; HM: hand movement

Patient	Sex and age at diagnosis in years	Systemic involvement	Initial manifestation	Ocular findings	Snellen (LogMAR) visual acuity OD	Snellen (LogMAR) visual acuity OS	Treatment		
							Steroids	Immunosuppressants	Biologics
1	F, 30	Cutaneous	Granulomatous dermatitis	Leukoma, pterygium	20/25 (0.10)	20/20 (0.00)	Oral prednisone	No	No
2	F, 52	Lymphatic (mediastinal)	Cough, dyspnea	Uveitis (anterior, non-granulomatous), KCS, pterygium	20/25-2 (0.14)	20/25-1 (0.12)	Oral prednisone	No	No
3	F, 78	Lymphatic (mediastinal), cardiac, cutaneous	Dyspnea	POAG^*^	None	None	Oral prednisone	No	No
4	F, 66	Pulmonary	Dyspnea	None	None	None	Oral prednisone	Azathioprine	No
5	F, 70	Lymphatic (mesenteric)	Abdominal pain	None	None	None	Oral prednisone	No	No
6	M, 68	Lymphatic (mediastinal)	Cough, dyspnea	KCS	20/25 (0.10)	20/25 (0.10)	Oral prednisone	No	No
7	M, 43	Lymphatic (mediastinal)	Ocular pain, photophobia	Dacryoadenitis, solitary choroidal granuloma, KCS	HM (2.40)	20/40 (0.30)	Intravenous and periocular methylprednisolone	MTX, Azathioprine	Adalimumab
8	F, 81	Ocular (panuveitis), cutaneous (granulomatous dermatitis),	Ocular pain, granulomatous dermatitis	Uveitis (anterior, non-granulomatous to panuveitis), periphlebitis, chorioretinal peripheral lesions, uveitic macular edema, cataracts, KCS	20/70 (0.54)	20/50 (0.40)	Topical (prednisolone acetate), periocular (methylprednisolone), intravitreal (dexamethasone implant)	MTX	No

The median follow-up duration was five years (range: 3-10 years. Two patients were followed for 10 years, one for eight years, two for five years, one for four years, and two for three years. All patients remained under medical surveillance due to chronic comorbid conditions. Notably, the two patients with ocular sarcoidosis continue to receive active treatment with immunosuppressants and ophthalmologic follow-up, currently reaching three years of continuous care.

## Discussion

The results of this 10-year retrospective study indicate that sarcoidosis in our population predominantly affects women, a finding consistent with previous reports in the literature [1-3,22]. However, epidemiological data from Latin America remain limited. In a Mexican single-center study, cutaneous involvement was reported in 42.8% of patients, while pulmonary disease occurred in 66.6%, a notably lower rate than observed in many international cohorts [18]. In contrast, pulmonary sarcoidosis was the most frequent subtype in our study (62.5%), followed by cutaneous and ocular involvement, each accounting for 25%. These differences suggest potential regional variability in clinical presentation or differences in referral and diagnostic patterns.

The mean age of onset in our cohort was 61 years, which is slightly higher than that reported in both international and national studies [1,3,18,19,22]. This may reflect a referral bias typical of tertiary care centers, where patients with more complex or long-standing disease are more likely to be seen. Additionally, delays in diagnosis due to nonspecific early symptoms or barriers in access to specialized care could contribute to a later age at presentation. Demographic or environmental factors specific to our region may also play a role and warrant further investigation.

Ocular sarcoidosis was found to be the second most frequent manifestation in our cohort (25%), along with cutaneous involvement. All patients with ocular disease eventually developed systemic sarcoidosis, in line with previous observations [1-3,22]. However, the true frequency of ocular involvement may be underestimated, as not all patients underwent ophthalmologic screening during follow-up.

Our findings can be contextualized by comparison with a Mexican case series of 14 patients with ocular sarcoidosis [19]. In that study, anterior uveitis (50%) and panuveitis (28.6%) were the most frequent manifestations, with granulomatous inflammation present in 28.6% of cases and dacryoadenitis in 14.3%. In contrast, KCS was observed in only 14.3% of patients. In our cohort, however, KCS was the most common ocular finding (50%), followed by uveitis (25%), a solitary choroidal granuloma (12.5%), and unilateral dacryoadenitis (12.5%). Notably, all cases of uveitis in our series were bilateral and non-granulomatous, which is considered atypical in the context of sarcoidosis. This contrasts with the previously reported series, where both granulomatous and non-granulomatous forms were documented [19]. Additionally, KCS may result from granulomatous infiltration of the lacrimal gland, which can occur without overt symptoms, potentially contributing to its increased detection in our study [17,19]. 

Although KCS was the most frequent ocular manifestation in our cohort, its interpretation requires caution due to its nonspecific nature and potential overlap with age-related dry eye or autoimmune conditions. Notably, one patient with KCS also had rheumatoid arthritis and presented with bilateral non-granulomatous anterior uveitis, highlighting the challenge of differentiating sarcoidosis-related ocular involvement from other systemic autoimmune diseases. This limitation underscores the need for comprehensive clinical evaluation and the use of standardized diagnostic criteria when assessing ocular sarcoidosis [20].

These discrepancies highlight the clinical heterogeneity of sarcoidosis across populations and emphasize the importance of applying standardized diagnostic frameworks, such as the IWOS criteria, especially for atypical ocular presentations. The progression from ocular manifestations to systemic disease observed in both our cohort and previous reports further supports the need for a multidisciplinary diagnostic approach [15,18,19]. Routine ophthalmologic screening in patients newly diagnosed with sarcoidosis may enhance the detection of subclinical or early ocular involvement.

Although serum ACE levels were available for only two patients, both showed elevated values (median 70.6 U/L). Given the very small number tested, these findings should be interpreted with caution, as ACE is not specific to sarcoidosis but can provide supportive diagnostic information when considered alongside clinical and radiological findings. According to WASOG diagnostic criteria, sarcoidosis requires a compatible clinical and radiological presentation, histological evidence of non-caseating granulomas, and the exclusion of alternative causes, especially infections [8].

In our cohort, infectious etiologies were systematically excluded, including tuberculosis, which was ruled out using the Purified Protein Derivative (PPD) test. However, it is important to note that BCG vaccination has been routinely administered in Mexico for decades, which can cause false-positive PPD results and limit its specificity. Nevertheless, one patient developed disseminated tuberculosis one year after starting adalimumab, highlighting the importance of thorough screening and ongoing monitoring for latent infections [15]. In tuberculosis-endemic regions, the use of more sensitive screening tools such as interferon-gamma release assays (IGRAs) should be considered before initiating biologic therapies [15].

Visual outcomes were mostly favorable, with only one case of severe visual impairment. This suggests that early detection, timely intervention, corticosteroid therapy, and multidisciplinary follow-up likely contributed to preserving vision in the majority of patients. However, it is important to acknowledge that only five of the eight patients underwent ophthalmologic assessment, potentially due to the absence of overt ocular symptoms in the remaining patients, which could underestimate the true burden of ocular involvement. Additional ocular findings, such as pterygium, primary open-angle glaucoma, and cataracts, were observed, which may result from the disease process itself or as adverse effects of corticosteroid treatment [15]. The presence of diverse comorbidities, including autoimmune, metabolic, and cardiovascular conditions, further underscores the need for a comprehensive, multidisciplinary approach to patient management to optimize both ocular and systemic outcomes [15].

All patients in our cohort received corticosteroid therapy, aligning with current clinical guidelines that establish corticosteroids as the cornerstone of sarcoidosis management [3,15]. Systemic corticosteroids (oral prednisone) were prescribed in 75% of cases, particularly those with multisystem involvement, emphasizing their role as first-line agents for systemic disease control. In contrast, 25% of patients were managed with topical ocular corticosteroids or periocular injections, reflecting a more localized therapeutic approach in cases with isolated ocular manifestations [11,15,22]. Immunosuppressive therapy was indicated in 75% of patients. Methotrexate was used in four patients (50%) as a steroid-sparing agent. Two patients (25%) were treated with azathioprine; one of them was initially on methotrexate and later switched to azathioprine due to intolerance. This treatment progression highlights the need for flexible and individualized management strategies for refractory or multisystemic sarcoidosis [11,15,22].

Notably, one patient with extensive multisystem disease required escalation to biologic therapy with adalimumab, in addition to corticosteroids and immunosuppressants. The use of biologics, especially TNF-α inhibitors, has become increasingly important in cases of refractory organ-threatening sarcoidosis, particularly for patients who experience adverse effects from corticosteroids or have contraindications to their use. However, administering these therapies requires careful screening and monitoring, as demonstrated by this patient's case of disseminated tuberculosis [11,15,22].

Overall, this small case series underscores the clinical heterogeneity of sarcoidosis and the relevance of ophthalmic involvement, especially when it presents initially. Although limited by sample size, our findings suggest a relatively high frequency of KCS and a later age at diagnosis compared to previous cohorts [1,3,18,19,22]. One severe case of tuberculosis reactivation following biologic therapy further highlights the need for vigilant infectious disease screening in endemic environments.

These observations support the importance of routine ophthalmologic evaluation at the time of diagnosis to detect subclinical ocular involvement. In tuberculosis-endemic regions, thorough pre-treatment screening using interferon-gamma release assays (IGRAs), in addition to or instead of the PPD skin test, may reduce the risk of reactivation. Use of standardized diagnostic tools for ocular surface evaluation, such as TBUT, Schirmer’s test, and ocular surface staining graded using validated systems like The Oxford Schema, may improve the recognition of KCS, an underrecognized yet potentially common ocular manifestation of sarcoidosis [21,22]. Interdisciplinary management remains essential for optimal care, especially in patients with systemic disease or comorbidities.

While the findings are not generalizable, they provide meaningful, region-specific insights and highlight the need for larger, prospective studies to better characterize the clinical spectrum of sarcoidosis in Latin America.

## Conclusions

Although limited by a small sample size, this study offers meaningful clinical and ophthalmologic observations in a Mexican sarcoidosis cohort. Ocular involvement, including KCS, non-granulomatous anterior uveitis, and choroidal granuloma, was observed even in patients without initial ocular symptoms, highlighting the importance of routine ophthalmologic screening in all sarcoidosis cases. Early identification and multidisciplinary management appeared to help preserve visual function in most patients. In regions with endemic infectious diseases and limited diagnostic resources, underrecognition remains a significant challenge. Further prospective studies are required to refine diagnostic strategies and optimize patient care in similar settings.
